# Can health service equity alleviate the health expenditure poverty of Chinese patients? Evidence from the CFPS and China health statistics yearbook

**DOI:** 10.1186/s12913-021-06675-y

**Published:** 2021-07-21

**Authors:** Shaoliang Tang, Ling Yao, Chaoyu Ye, Zhengjun Li, Jing Yuan, Kean Tang, David Qian

**Affiliations:** 1 School of Health Economics and Management, Nanjing University of Chinese Medicine, Nanjing, China; 2 Faculty of Science, Skane, Lund University, Lund, Sweden; 3 Swinburne Business School, Swinburne University of Technology, Melbourne, Australia

**Keywords:** Health service equity, Health expenditure poverty, CFPS, Elastic net regression

## Abstract

**Objectives:**

To comprehend the relationship between various indicators of health service equity and patients’ health expenditure poverty in different regions of China, identify areas where equity in health service is lacking and provide ideas for improving patients’ health expenditure poverty.

**Method:**

Data from China Family Panel Studies (CFPS) in 2018 and the HFGT index formula were used to calculate the health expenditure poverty index of each province. Moreover, Global Moran’s I and Local Moran’s I test are applied to measure whether there is spatial aggregation of health expenditure poverty. Finally, an elastic net regression model is established to analyze the impact of health service equity on health expenditure poverty, with the breadth of health expenditure poverty as the dependent variable and health service equity as the independent variable.

**Results:**

In the developed eastern provinces of China, the breadth of health expenditure poverty is relatively low. There is a significant positive spatial agglomeration. “Primary medical and health institutions per 1,000 population”, “rural doctors and health workers per 1,000 population”, “beds in primary medical institutions per 1,000 population”, “proportion of government health expenditure” and “number of times to participate in medical insurance (be aided) per 1,000 population” have a positive impact on health expenditure poverty. “Number of health examinations per capita” and “total health expenditure per capita” have a negative impact on health expenditure poverty. Both effects passed the significance test.

**Conclusion:**

To enhance the fairness of health resource allocation in China and to alleviate health expenditure poverty, China should rationally plan the allocation of health resources at the grassroots level, strengthen the implementation of hierarchical diagnosis and treatment and encourage the investment in business medical insurance industry. Meanwhile, it is necessary to increase the intensity of medical assistance and enrich financing methods. All medical expenses of the poorest should be covered by the government.

**Supplementary Information:**

The online version contains supplementary material available at 10.1186/s12913-021-06675-y.

## Introduction

Over the past few decades, the issue of equity in health services has attracted great attention. The World Report states that equity is at the heart of primary health care. Health service refers to the general term of social medical treatment, public health activities, facilities and systems. It includes health institutions with sound services for health promotion, disease prevention, treatment and rehabilitation, a complete and quality-assured service network, a certain amount of economic input, fair and reasonable allocation of health resources, and guaranteed service availability [1]. Health service equity means that efforts should be made to reduce the unfairness and undue social gaps in the use of health and health services and strive to make every member of society meet basic living standards. It includes but is not limited to the equity of health care, the equity of health and the equity in the financing of health services [2].

Scholars’ studies on health service equity have mainly focused on the differences in health service equity [3–5], influencing factors [6–8], policy research [9–11] and social influencing factors [12–15]. In addition, they have also done much research on the evaluation criteria and measurement of health service equity. John W. Peabody (1996) et al. believed that a fair system should guarantee the poor and other vulnerable groups access to basic medical services [16]. Liburd Leandris C (2020) et al. supposed that government public health plays a core role in promoting the realization of the highest level of health for all people, including seeking fair access to high-quality health facilities, drugs, commodities and services. All functional departments of the public health system can strengthen cooperation to increase the opportunity for citizens to obtain the best health [17]. Xueshan Sun (2018) et al. measured health equity from the perspective of health demand and medical seeking behavior [18]. Douglas C. Dover (2019) et al. proposed a framework for measuring and monitoring health equity, including the health policy context, health-related behaviors and beliefs, stress, quality of care and health care utilization [19]. The Working Group for Monitoring Action on the Social Determinants of Health (2018), funded by the World Health Organization, Public Health Agency of Canada, and the Canadian Institutes for Health Research, developed a set of core indicators for government action on the social determinants of health. The indicators are based on five areas of action of the Rio de Janeiro Political Declaration, including the public health and social security level, system comprehensive service coverage level and 36 other targets [20]. According to the characteristics of China’s new rural cooperative medical system, Dongqing Ye (2016) insisted that health service equity should include the fairness of health financing, utilization and health and medical assistance [21]. Rosalind McCollum (2019) et al. assessed the perceived equity of health services by health system and community level actors in Kenya. They believed that for Kenya to achieve universal health coverage, the government must address all aspects of equity, including quality issues, in which community health services can play a key role in achieving health equity [22]. Wei Lang (2016) et al. found that climate change is a serious reason for the spatial inequality of access to health care for vulnerable groups. To ensure health service equity in the San Francisco Bay Area, they advised redistributing public health resources geographically and putting more effort into vulnerable groups [23]. Rezapour A (2014) measured the medical payment ability of families based on questionnaire survey data and calculated the equity index of medical care financing. The results show that the medical care financing system is not equal, and health financing should start by protecting the needs of low-income families for health expenditure to improve the equity of health [24]. In summary, although scholars had different statements on the criteria for judging the fairness of health services, they can generally be summarized into five aspects: health equity, health service utilization equity, health financing equity, health resource distribution equity and vulnerable group equity.

In recent years, as China’s health care costs have risen sharply, low-income residents have been unable to obtain the care they need, with the result of falling into health expenditure poverty. Scholars have studied the influencing factors of health expenditure poverty from different aspects, mainly including the medical insurance system, medical assistance level, equity of health resources and community health service level. Yang Zhao, Brian Oldenburg (2020) et al. studied the trends and socioeconomic differences of catastrophic health expenditure (CHE) and health poverty after China’s major health system reform and discussed the impact of chronic diseases on CHE and health poverty [25]. Arigbeshola (2018) used data from the Nigerian Living Standard Survey to evaluate the disastrous and impoverished impact of health payments on Nigerian families, and the study revealed that the government urgently needs to increase investment in public health care funds [26]. Ahmed s. (2018) utilized the Vietnamese family life standard survey data to check the geography, health systems, environment and population variables related to health care expenditures of catastrophic health expenditure by a multiple logistic regression model. The results indicated that Vietnam’s health insurance program financial support capability should be improved and expanded to reduce the poverty caused by health care payments, especially in the Mekong Delta region [27]. Jia Xie (2018) studied health expenditure poverty using catastrophic health expenditure and health expenditure poverty. She discovered that the medical insurance system alleviated the health expenditure poverty of rural residents in China to a certain extent, but the supporting capacity was weak, and medical assistance should be the focus of system construction [28].

Medical and health service utilization is closely related to population health outcomes [29]. However, how is the relationship between health service utilization and health expenditure poverty? Can the improvement of equity in health services effectively alleviate health expenditure poverty in groups? At present, most studies have dissociated health expenditure poverty from health service equity, but there are few studies on the link between the two. Therefore, this paper innovatively combined health expenditure poverty with health service equity to explain the above problems. We first explored the current situation of health expenditure poverty in various regions of China and then studied the impact of health service equity on the health expenditure poverty of residents in various regions. Finally, we propose feasible policy suggestions to provide ideas for improving the fairness of health services in China and alleviating the health expenditure poverty of patients.

## Method

### Data sources

The data of independent variables in this paper came from the China Health Statistics Yearbook 2019, and the data of dependent variables came from the survey results of China Family Panel Studies (CFPS) conducted by the Chinese Social Science Survey Center (ISSS) of Peking University in 2018 [30]. The CFPS is a national comprehensive family social tracking survey. It focuses on economic and non-economic benefits dimension of investigation on residents, involving economic behavior, education achievements, family relationship and dynamics, population migration, the physical and mental health, and many other research topics. Through tracking and collecting data at individual, household and community levels to reflect changes in China’s society, economy, population, education and health, the CFPS provides data support for academic research and public policy analysis. The survey objects were all family members and sample families in 25 provinces, autonomous regions and municipalities (excluding Taiwan, Hong Kong and Macao) of China, with a total sample size of 14,241 families and 32,669 individuals [31].

### Independent variables

According to the literature analysis in the introduction, we know that the academic community has put forward a variety of standards to measure health service equity, which can be summarized into five aspects: health equity, health services utilization equity, health financing equity, health resources distribution equity and equity of vulnerable groups. We believe that if the standard is too simple, the complexity of the fairness issue is not conducive to understanding. If it is too complex to operate realistically, fairness is even harder to guarantee. Therefore, health service utilization equity, health financing equity, health resource distribution equity and the equity of vulnerable groups were used as the measurement criteria of health service equity in this paper, in line with the status quo of China’s health service system and the availability of data. Specifically, we used four dimensions of health resource allocation, health service utilization, health financing and medical assistance as the primary indicators to measure health service equity. In view of China’s regional differences in such aspects as economy and population scale, we wielded the data of regional population and primary index above to calculate 20 secondary indicators for the sake of sample scientificity. Finally, we established the evaluation system of health service equity in this paper (Table 1) and took this system as the main independent variable. This approach not only combines the current main research results extensively, but also ensures the simplicity and scientificity of the evaluation criteria.

**Table 1:** Evaluation index system of health service equity

Primary indicators	Secondary indicators	Abbreviation
Allocation of health resources	Hospitals per 1000 population	*X*_1_
	Primary medical and health institutions per 1000 population	*X*_2_
	Professional public health institutions per 1000 population	*X*_3_
	Health technicians per 1000 population	*X*_4_
	Rural doctors and health workers per 1000 population	*X*_5_
	Hospital beds per 1000 population	*X*_6_
	Beds in primary medical institutions per 1000 population	*X*_7_
	Beds in specialized public health institutions per 1000 population	*X*_8_
Utilization of health services	Number of outpatient patients per capita	*X*_9_
	Number of health examinations per capita	*X*_10_
	Number of hospitalizations per capit	*X*_11_
	Annual hospitalization rate of residents	*X*_12_
Health financing	Proportion of government expenditure on health	*X*_13_
	Proportion of social health expenditure	*X*_14_
	Proportion of personal health expenditure	*X*_15_
	Total health expenditure per capita	*X*_16_
Medical assistance	Number of times to participate in medical insurance (be aided) per 1000 population	*X*_17_
	Direct medical assistance per 1000 people	*X*_18_
	Subsidies to participate in medical insurance expenses per 1000 population	*X*_19_
	Direct medical assistance expenditure per 1000 population	*X*_20_

### Dependent variable

Poverty is a complex global problem. Studies on this topic have become a hot spot in academic circles. The measurement of poverty is an indispensable way to study poverty. In this regard, scholars have also made many efforts. Initially, scholars mainly used the basic poverty index to measure the poverty degree. They measured the poverty incidence rate and poverty gap in a statistical sense. Nevertheless, the results were often quite disparate from the intuitive feeling. Then, Sen (1976) introduced the poverty axiom system and built the Sen poverty index formula [32]. Thanks to the efforts of Sen and subsequent scholars, research on the axiomatic poverty index has been improved, with the emergence of the SST poverty index [33, 34] and Foster-Greer-Thorbecke (FGT) poverty index [35]. The SST index, which has the greatest advantage of being within the interval of [0,1], still cannot satisfy all the axioms. The FGT poverty index has the superiority of decomposability, which can not only reflect the breadth of poverty distribution, but also measure the depth and strength of poverty distribution. This feature remedies for the defects and deficiencies of the poverty incidence and poverty gap index, making the FGT poverty index widely adopted by some scholars and local government agencies in poverty measurement. The FGT poverty index is expressed as follows:

1 $$ FG{T}_{\alpha }=\frac{1}{n}{\sum}_{i=1}^q{\left(\frac{\mathrm{z}-{\mathrm{y}}_i}{z}\right)}^{\alpha } $$

where *n* is the total number of residents in each region, *q* is the total number of poor residents in each region, *y*_*i*_ is the income level of the *ith* individual, *z* is the poverty line, and *α* is the coefficient of poverty aversion [36, 37]. The larger the value of parameter *α* is, the higher the degree of aversion to poverty is, and the greater the weight given to the poor with lower income level is. When *α* = 0, *FGT*(0) represents the Headcount Index of poverty, which measures the proportion of poor people in the total surveyed population and reflects the breadth of poverty. When *α* = 1, *FGT*(1) represents the poverty gap index, which measures the funds needed by the surveyed poor people to eliminate poverty and reflects the depth of poverty. When *α* = 2, *FGT*(2) represents the squared poverty gap index, which is the weighted poverty gap rate, namely the sum of squares of the income level of each poor population and the gap between the poverty line [38], reflecting the strength of poverty.

2 $$ {HFGT}_{\alpha }=\frac{1}{n}\sum \limits_{i=1}^q{\left(\frac{z-{y}_i}{z}\right)}^{\alpha } $$

Based on the concept of the FGT index, combined with the research of some scholars [39, 40], this paper constructed an expression to measure health expenditure poverty (formula 2), where *n* is the total number of residents in each region, *q* is the total number of residents living in poverty due to health expenses, *y*_*i*_ is the income level of the *ith* resident living in poverty due to health expenses, *z* is the poverty line, and *α* is the coefficient of poverty aversion. The meaning of *α* is the same as formular 1.

China’s poverty alleviation standard is the 2011 constant price of farmers’ per capita annual income of RMB 2300, so this paper first selected RMB 2300 as the poverty line *q* to calculate health expenditure poverty index. We also calculated the new poverty line as RMB 2648.56 according to China’s 2012–2018 CPI index to test robustness and carried out the elastic net regression analysis.

This paper assumes that if nonpoor residents fall below the poverty line because of health expenditure, they suffer from health expenditure poverty. Health expenditure excludes the part that has been reimbursed and that is expected to be reimbursed. We screened “total household income in the past 12 months” and “total health expenditure in the past 12 months” in the 2018 CFPS questionnaire. After deleting data with too many missing values and outliers, 12,079 samples were retained on aggregate. Among them, the number of residents caught in health expenditure poverty was 1529. Finally, we calculated the health expenditure poverty index for 28 regions in China as the dependent variable.

### Research methods

#### Ridge, lasso and elastic net regression

It is well known that OLS methods tend to lead to overfitting of the model. This problem is more serious when there are many predictive variables and high collinearity between variables [41–43]. Overfitting models tend to include unnecessary redundant variables, overestimate the role of some predictors, and weaken the simplicity of the model [42]. With the rapid development of the machine learning field, an increasing number of statistical tools have emerged to offset the limitations of traditional methods. Regularization methods represented by lasso (least absolute shrinkage and selection operator) regression [44] and ridge regression methods can effectively optimize OLS estimation and deal with overfitting problems [44–47]. Regularization controls the complexity of the model by adding some penalty items or constraint conditions to reduce overfitting. Lasso regression and ridge regression are *l*_1_ regularization and *l*_2_ regularization of linear regression, respectively. Their regression models are as follows:

3 $$ {\hat{\beta}}_{lasso}=\mathit{\arg}\ {\mathit{\min}}_{\beta \in {R}^p}\frac{1}{2}{\left\Vert y- X\beta \right\Vert}_2^2+\lambda {\left|\left|\beta \right|\right|}_1 $$

4 $$ {\hat{\beta}}_{ridge}=\mathit{\arg}\ {\mathit{\min}}_{\beta \in {R}^p}\frac{1}{2}{\left\Vert y- X\beta \right\Vert}_2^2+\lambda {\left|\left|\beta \right|\right|}_2^2 $$

where $\frac{1}{2}{\left|\left|y- X\beta \right|\right|}_2^2$ is the loss function of the regression model. *λ*||*β*||_1_ uses the *l*_1_ norm as the regularization penalty function, and $\lambda {\left|\left|\beta \right|\right|}_2^2$ uses the *l*_2_ norm as the regularization penalty function. As the regularization term of punishment, the *l*_1_ norm has a stronger capacity to sparse regression coefficient vector and can compress the estimated coefficient of the redundant predictor variable to 0, which plays the role of variable screening at the same time as the compression coefficient. Therefore, it can effectively avoid the problem of insufficient generalization ability of the model caused by overfitting and obtain a simpler model with higher prediction efficiency [48]. Compared with ridge regression, lasso regression has a stronger function of variable selection and has the function of dimension reduction in high-dimensional space. However, the drawback of Lasso regression is that it tends to choose one from a group of related variables, which tends to cause other variables to be ignored and the predictive power of the model to be reduced.

5 $$ {\mathit{\min}}_{\beta_{0,}\beta \in {R}^{p+1}}\left[\frac{1}{2N}{\sum}_{i=1}^N{\left({y}_i-{\beta}_0-{x}_i^T\beta \right)}^2+{\lambda P}_{\alpha}\left(\beta\ \right)\right] $$

6 $$ {P}_{\alpha}\left(\beta \right)=\left(1-\alpha \right)\frac{1}{2}{\left\Vert \beta \right\Vert}_{l_2}^2+\alpha {\left\Vert \beta \right\Vert}_{l_1} $$

Formulas 5 and 6 demonstrate the principle of elastic net regression. In formula 5, *λP*_*α*_(*β* ) is the penalty term. *λ* is the penalty parameter and *λ* ≥ 0. When *λ* = 0, it means no penalty. While *λ* = ∞, it represents a complete penalty. *α* controls the proportion of ridge regression and lasso regression components and 0 ≤ *α* ≤ 1. When *α* = 0, Ridge regression will be performed. If *α* = 1, lasso regression will be performed. Elastic net regression dynamically combines lasso regression and ridge regression and simultaneously uses the *l*_1_ norm and *l*_2_ norm to select variables, which improves the accuracy of the model [49]. Therefore, this paper chooses elastic net regression as the main regression analysis method.

#### Global Moran’s I and local Moran’s I test

The first law of geography points out that everything is related to other things, and similar things are more closely related [50]. Spatial autocorrelation analysis characterizes the degree of correlation between geographical samples. If the measured values are increasingly similar with the decrease in the distance between samples, the relationship between samples is positively correlated. Similarly, if the otherness increases as the distance between samples decreases, there is a negative spatial correlation. Furthermore, if the measured value does not change with the change in the distance between samples, it is a spatial random distribution. Spatial autocorrelation analysis changes the spatial autocorrelation analysis of geographic data from qualitative to quantitative description through the calculation of the spatial autocorrelation index [51]. In this paper, global Moran’s I and Local Moran’s I test were used for spatial autocorrelation analysis. The principle of Global Moran’s I and local Moran’s I tests is put into the appendix in consideration of space limits.

## Spatial autocorrelation analysis

Spatial aggregation analysis can better explain the spatial distribution characteristics of health expenditure poverty and the influence degree of related socioeconomic factors. There are some differences in health expenditure poverty among different regions in China, and it is necessary to judge whether there is spatial autocorrelation. In this paper, global Moran’s I and local Moran’s I tests were used to inspect the spatial correlation.

### Results of global Moran’s I test

**Fig. 1:**
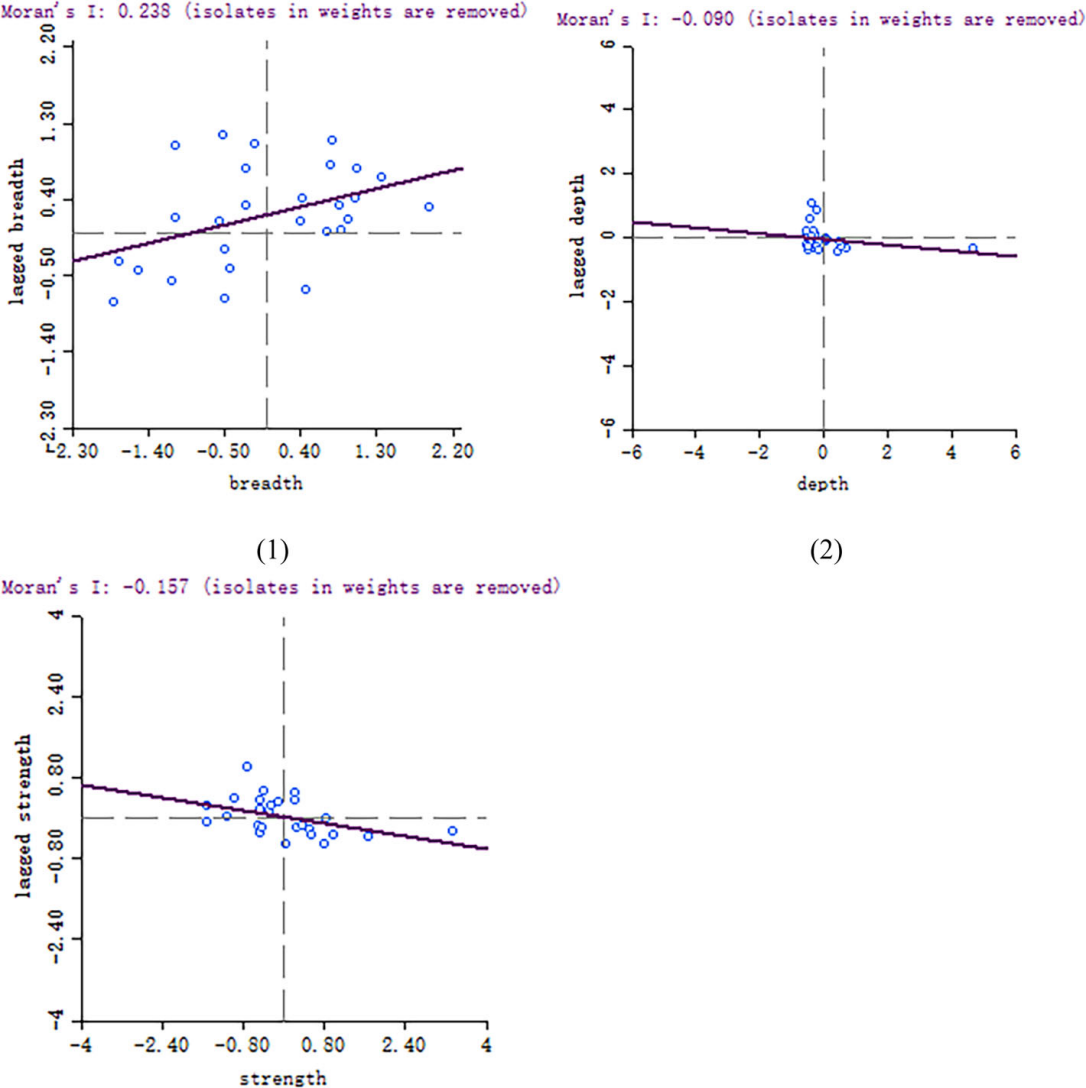
Moran scatter plot of the breadth, depth and strength of health expenditure poverty in different regions of China

We calculated the global Moran’s I for the breadth, depth and strength of health expenditure poverty across China and tested the hypothesis of its spatial correlation. We drew the Moran scatter plot of the breadth, depth and strength of health expenditure poverty in various regions of China in 2018 to directly reflect the local spatial characteristics of health expenditure poverty in various regions of China (Fig. 1).

As shown in Fig. 1, in the Moran scatter plot of the breadth of health expenditure poverty, the scattered points are mainly distributed in the first and third quadrants, representing that the “H-H” and “L-L” clusters are more than the “L-H” and “H-L” clusters. That is, the breadth of health expenditure poverty in China has a strong positive spatial correlation. The regions with higher (lower) breadth of health expenditure poverty are more likely to produce clustering, and these regions have less spatial heterogeneity. In addition, we can see that the scattered points of the strength of health expenditure poverty are mainly distributed in the second and fourth quadrants, whereas the distribution of scatterplots in each quadrant is not uniform in the Moran scatter plot of the depth. The results above show that the strength of health expenditure poverty in China has a strong negative spatial correlation. The regions with higher and lower strengths of health expenditure poverty in China are more likely to produce clustering, and these regions have large spatial heterogeneity. Again, we can preliminarily conclude that the spatial correlation of the depth of health expenditure poverty in different regions of China is weak.

Table 2 reports the global Moran’s I and its significance test results of health expenditure poverty in 28 regions of China calculated by Geoda software. Among them, the global Moran’s I of health expenditure poverty breadth in 28 regions of China is positive. The *p*-value is 0.021, less than 0.05, that is, it is significant at the 5% level. This reveals that there is a significant positive spatial correlation between the breadth of health expenditure poverty in different regions of China. This may be the result of regional trends in the level of economic development in different regions of China. The level of economic development in the eastern coastal areas is generally higher than that in the inland and western regions, which may make a positive spatial aggregation between the breadth of health expenditure poverty come into being. However, the level of 5% indicates that the spatial correlation between the depth and strength of health expenditure poverty is not significant. We believe that this may be caused by the heterogeneity of economic development, medical security level and government poverty alleviation policies in different regions of China. The depth of health expenditure poverty reflects the funds needed by the surveyed population to eliminate poverty caused by health expenditure, and the strength of health expenditure poverty reflects the square sum of the gap between the income level and the poverty line of the average poor population caused by health expenditure. China has a vast territory, and due to the great differences in economic development and medical security levels among different regions, the income gap of residents in different regions is different, which leads to the lack of obvious aggregation of the depth and strength of health expenditure poverty. In particular, since the implementation of the Targeted Poverty Alleviation Strategy in 2014, China has made national efforts to promote poverty alleviation, and formulated more than 30 policy tools, including poverty alleviation through health, talent, society, education and culture. Taking the health poverty alleviation policy as an example, the Chinese government has made the new rural medical cooperation and serious disease insurance system more favorable to the poor population, increased medical assistance, temporary and charity assistance, and designed a series of medical rehabilitation projects for the disabled in rural areas. These practices have greatly reduced the poverty alleviation “gap” of the poor, which may be one of the important reasons why the spatial correlation between the depth and strength of health expenditure poverty in different regions is not significant.

**Table 2:** Global Moran’s I and its significance test results of health expenditure poverty in various regions of China

Indicators	Global Moran’s I	Z-value	***P***-value
Breadth of health Expenditure poverty	0.238	2.0969	0.021
Strength of health expenditure poverty	−0.157	−1.0096	0.147
Depth of health expenditure poverty	−0.090	−0.7398	0.219

### Results of local Moran’s I test

**Fig. 2:**
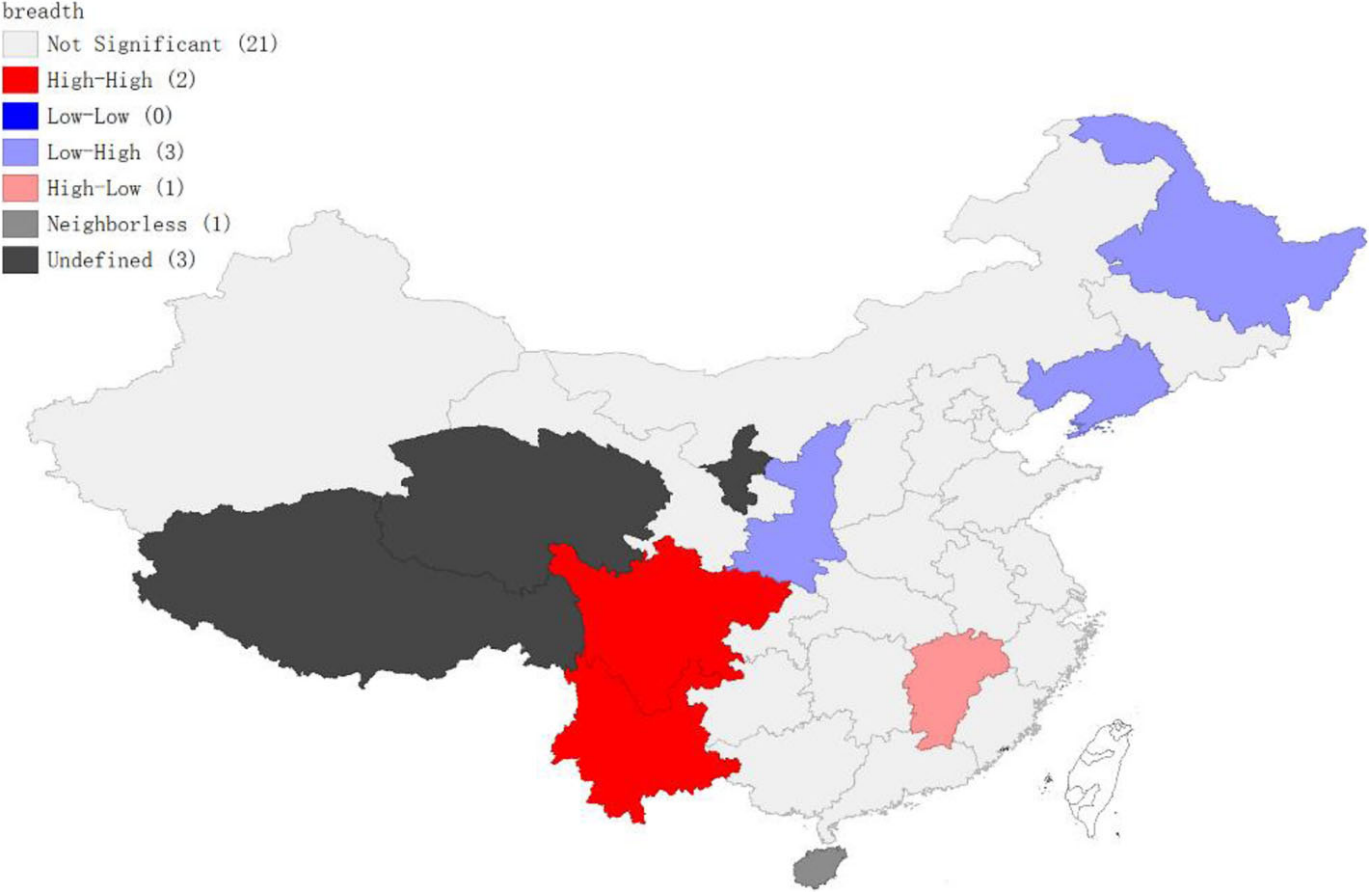
Local Moran’s spatial agglomeration map of health expenditure poverty breadth in China

Based on the autocorrelation of global Moran’s I, we used local Moran’s I test to define the specific location of spatial autocorrelation features to overcome the limitation of not being able to observe the specific location of clustering similar regions. The global Moran’s I of the breadth of health expenditure poverty mentioned above passed the significance test. We further explored the spatial correlation of the breadth of health expenditure poverty in different regions of China through the local Moran’s I (Fig. 2), and a significance test was conducted (Table 3).

**Table 3:** Local Moran’s I test result of health expenditure poverty breadth in partial provinces of China

Province	LISA_I	LISA_CL	P-Value
Heilongjiang	−0.595	3.000	0.045
Jiangxi	−0.303	4.000	0.046
Liaoning	−0.155	3.000	0.040
Shaanxi	−0.194	3.000	0.022
Sichuan	0.829	1.000	0.032
Yunnan	0.833	1.000	0.045

As shown in Fig. 2, the breadth of health expenditure poverty in China is mainly concentrated in the northeast and southwest, and only a small part is concentrated in the inland areas. Among them, the breadth of health expenditure poverty in Yunnan and Sichuan provinces shows an “H-H” cluster, which indicates that the breadth of health expenditure poverty in these two regions is high, so are the surrounding areas. The breadth of health expenditure poverty in Heilongjiang, Liaoning and Shaanxi provinces displays an “L-H” type aggregation, indicating that the breadth of health expenditure poverty in these provinces is low, while their surrounding areas, on the other hand, do the opposite. The breadth of health expenditure poverty in Jiangxi Province shows an “H-L” type aggregation. This means that the breadth of health expenditure poverty in Jiangxi Province is higher, while its surrounding areas is lower. The results are basically in line with China’s economic development.

Table 3 reports the local Moran’s I test results for three aggregation types. The results show that the local Moran’s I of the three types of aggregation passed the significance test.

## Regression analyses

### Independent variable collinearity test

The diagnostic indicators of collinearity include tolerance and variance inflation factor (VIF), and the two judgments basis are mutually reciprocal. Specifically, the model is proven to have multiple collinear phenomena in a strict sense when the tolerance is less than 0.1 or *VIF* > 10. However, in the diagnosis of multicollinearity, it is found that the VIFs of most variables are close to or far greater than 10, except *X*_6_, *X*_18_ and *X*_20_ which are relatively small. In addition, the significance test shows that all explanatory variables failed the significance test (Table 4). Serious multicollinearity is proven to exist between the variables. This justifies the use of elastic net regression to eliminate multicollinearity between variables.

**Table 4:** Collinearity test results of independent variables

Model	Unstandardized Coefficients		Standardized Coefficients	t	Sig.	95.0% Confidence Interval for B		Correlations			Collinearity Statistics	
	B	Std. Error	Beta			Lower Bound	Upper Bound	Zero-order	Partial	Part	Tolerance	VIF
Constant	−.005	.307		−.015	.988	−.712	.703					
*X*_1_	1.610	4.764	.210	.338	.744	−9.375	12.595	.238	.119	.052	.060	16.543
*X*_2_	.057	.110	.244	.519	.617	−.197	.311	.691	.181	.079	.106	9.434
*X*_3_	1.344	4.349	.239	.309	.765	−8.685	11.372	.318	.109	.047	.039	25.646
*X*_4_	.019	.029	.439	.672	.520	−.047	.085	−.407	.231	.103	.055	18.298
*X*_5_	−.005	.084	−.025	−.056	.956	−.198	.188	.714	−.020	−.009	.123	8.106
*X*_6_	.000	.002	.027	.107	.917	−.004	.004	.165	.038	.016	.370	2.700
*X*_7_	.098	.088	.703	1.118	.296	−.104	.300	.536	.368	.171	.059	16.946
*X*_8_	−.044	.278	−.061	−.158	.878	−.685	.597	.349	−.056	−.024	.158	6.323
*X*_9_	1.640E-5	.000	.651	.663	.526	.000	.000	−.590	.228	.101	.024	41.416
*X*_10_	.000	.000	−.563	−.969	.361	−.001	.000	−.513	−.324	−.148	.069	14.462
*X*_11_	−.001	.001	−.396	−.805	.444	−.003	.001	.206	−.274	−.123	.096	10.385
*X*_12_	−57.232	82.741	−.283	−.692	.509	− 248.032	133.569	−.062	−.238	−.106	.139	7.189
*X*_13_	.004	.004	.386	.867	.411	−.006	.013	.588	.293	.132	.118	8.485
*X*_15_	−.004	.005	−.296	−.716	.494	−.015	.008	.316	−.246	−.109	.137	7.300
*X*_16_	−1.808E-5	.000	−.627	−1.064	.319	.000	.000	−.623	−.352	−.162	.067	14.903
*X*_17_	−5.482E-5	.001	−.040	−.047	.964	−.003	.003	.474	−.017	−.007	.032	31.421
*X*_18_	1.547E-5	.000	.010	.040	.969	−.001	.001	−.167	.014	.006	.381	2.625
*X*_19_	−.007	.113	−.030	−.060	.954	−.267	.254	.237	−.021	−.009	.092	10.825
*X*_20_	.011	.022	.148	.511	.623	−.040	.063	.130	.178	.078	.278	3.598

### Elastic net regression analysis

We took the four dimensions of health service equity and the breadth of health expenditure poverty as independent and dependent variables. Then, we established the elastic net regression model by R software.

**Fig. 3:**
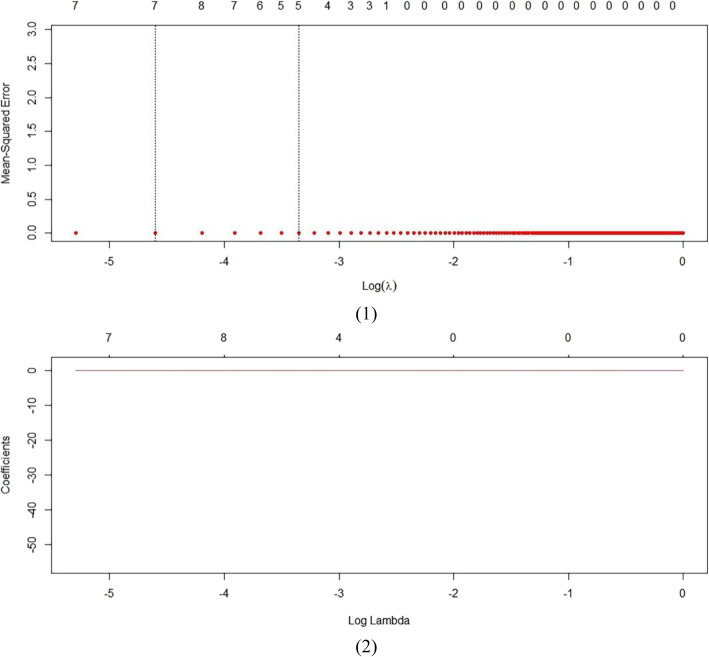
Elastic Net cross-validation regression results

Figure 3 shows the effect of the elastic net regression model when different parameters *λ* are used in cross-validation. Figure 3 shows that as the value of the parameter *λ* increases, the number of independent variables used in elastic net regression decreases, as does the prediction error of the model. The parameter *λ* that minimizes the model mean square error is 0.01005025. After obtaining the optimal parameters *λ*, we used the training data set to retrain the new elastic net regression model (Table 5). We also performed lasso and ridge regression analysis in R software to better verify the effect of elastic net regression. The results are presented in Table 5.

**Table 5:** Lasso, ridge and elastic net regression results

Variables	Regression coefficient		
	Lasso (*α* = 1)	Ridge ( *α* = 0)	Elastic Net regression (*α* = 0.5)
*X*_1_	–	4.508819e-02	–
*X*_2_	7.708620e-02	3.379657e-01	7.110331e-02
*X*_3_	–	4.499783e-03	–
*X*_4_	–	3.548136e-01	–
*X*_5_	2.467723e-02	7.395202e-02	2.676738e-02
*X*_6_	–	1.564979e-02	–
*X*_7_	5.074746e-03	4.111173e-01	7.995698e-03
*X*_8_	–	−7.187830e-02	–
*X*_9_	–	3.018772e-01	–
*X*_10_	−4.817413e-05	−3.708596e-01	−5.939468e-05
*X*_11_	–	−2.457680e-01	–
*X*_12_	–	−1.592871e-01	–
*X*_13_	2.376937e-03	2.695623e-01	2.233790e-03
*X*_14_	–	−2.250927e-02	–
*X*_15_	–	−2.623920e-01	–
*X*_16_	−1.478263e-06	−4.830314e-01	−1.781413e-06
*X*_17_	–	1.432623e-01	3.840092e-05
*X*_18_	–	2.432101e-02	–
*X*_19_	–	−3.505073e-02	–
*X*_20_	–	8.017608e-02	–
Intercept	9.079613e-04	−1.418397e-16	7.593978e-03

### Results

In ridge regression, we find that there is no independent variable with a regression coefficient of 0. Variables are not filtered very well by the ridge regression model. Although Lasso regression eliminated most of the insignificant variables, it did not have an effect on the dimensions of medical assistance (*X*_17_, *X*_18_, *X*_19_ and *X*_20_). Unsurprisingly, elastic net regression is a good complement to this.

The elastic net regression results show that the regression coefficients of 12 dependent variables equal to 0: *X*_1_, *X*_3_, *X*_4_, *X*_6_, *X*_8_, *X*_9_, *X*_11_, *X*_12_, *X*_14_, *X*_15_, *X*_18_, *X*_19_ and *X*_20_. This means that the model removes the features that have no significant impact on the dependent variable. Variables *X*_2_, *X*_5_, *X*_7_, *X*_10_, *X*_13_, *X*_16_ and *X*_17_ pass the screening, indicating that “primary medical and health institutions per 1,000 population”, “rural doctors and health workers per 1,000 population”, “beds in primary medical institutions per 1,000 population”, “number of health examinations per capita”, “proportion of government expenditure on health”, “total health expenditure per capita” and “number of times to participate in medical insurance (be aided) per 1,000 population” pass the significance test. This indicates that their impact on health expenditure poverty is statistically significant.

Specifically, “number of health examinations per capita” and “total health expenditure per capita” have a negative impact on health expenditure poverty. We believe that the improvement of people’s living conditions and health awareness has greatly promoted their demand for medical examinations, which can effectively prevent the occurrence of serious illnesses and reduce people’s high medical expenditures derived from severe disease; thus, health expenditure-induced poverty at the societal level will be lower. The greater the total health expenditure per capita is, the more and better health protection people can obtain correspondingly, which can also effectively prevent the risk of poverty due to illness, and people’s health expenditure is correspondingly lower. In contrast, “Primary medical and health institutions per 1,000 population”, “rural doctors and health workers per 1,000 population”, “beds in primary medical institutions per 1,000 population”, “proportion of government expenditure on health” and “number of times to participate in medical insurance (be aided) per 1,000 population” have a positive impact on health expenditure poverty. In this study, the health resources of regions with a higher level of economic development tend to be concentrated in general hospitals, which forces allocation level of health resources at the grassroots level to be low. As a result, in undeveloped areas, the more primary medical and health institutions per 1000 population, rural doctors and health workers per 1000 population and beds in primary medical institutions per 1000 population are, the easier residents fall into health expenditure poverty on account of their lower income levels.

### Robustness test

To verify the robustness of the model, we calculated the poverty line of 2018 based on China’s 2012–2018 CPI index. The new poverty line is RMB 2648.56. At the same time, we set “government health expenditure per capita” as the independent variable *X*_13_. Finally, we reintroduced the new poverty line and independent variable into the elastic net regression equation and obtained model 2 (Table 6).

**Table 6:** Robustness test results

Variables	Regression coefficient	
	Model 1 (*α* = 0.5)	Model 2 (*α* = 0.5)
*X*_1_	–	–
*X*_2_	7.110331e-02	7.077443e-02
*X*_3_	–	–
*X*_4_	–	–
*X*_5_	2.676738e-02	3.448887e-02
*X*_6_	–	–
*X*_7_	7.995698e-03	–
*X*_8_	–	–
*X*_9_	–	–
*X*_10_	−5.939468e-05	−4.327471e-05
*X*_11_	–	–
*X*_12_	–	–
*X*_13_	2.233790e-03	–
*X*_14_	–	−1.093319e-06
*X*_15_	–	−1.830648e-05
*X*_16_	−1.781413e-06	–
*X*_17_	3.840092e-05	7.725741e-05
*X*_18_	–	–
*X*_19_	–	–
*X*_20_	–	–
Intercept	7.593978e-03	9.623387e-02

As seen from Table 6, no significant differences exist between the results of model 1 and model 2. The variables screened by the model are relatively stable in general. Their coefficients fluctuate only within a small range. Therefore, we believe that the model in this paper is robust. Furthermore, we find that the proportion of personal health expenditure has a significant negative effect on health expenditure poverty. This may be caused by the characteristics of the medical behaviour of the high-income and low-income groups. High-income groups prefer to pursue high-quality medical care, which increases personal health expenditure. Some poor elderly people with major illnesses choose to forgo treatment. The choices of the two groups lead to higher personal health expenditure, along with low health expenditure poverty.

## Discussion

This article used data from the China Household Survey (CFPS) organized by the Chinese Social Science Survey Center of Peking University and the China Health Statistic Yearbook in 2018 to study the relationship between health service equity and health expenditure poverty. This paper finds that developed eastern provinces in China have a lower breadth of health expenditure poverty. Health expenditure poverty in Gansu, Guizhou, Sichuan, Yunnan and other regions is relatively high, raking low among 28 regions in China. Empirical results show that the primary indicators “health resource allocation”, “health service utilization” and “health financing” have a significant impact on health expenditure induced poverty. Among them, *X*_10_ and *X*_16_ have a significant negative impact on health expenditure poverty. *X*_2_, *X*_5_, *X*_7_, *X*_13_ and *X*_17_ have a significant positive impact on health expenditure poverty.

We find an interesting phenomenon. Generally, the higher the proportion of government health expenditure is, the lower the health expenditure of residents [52] is and the lower the health expenditure poverty is. However, the empirical results show that the higher the proportion of government expenditure on health is, the higher the health expenditure poverty is. We propose three possible reasons. First, there is an imbalance between regions and populations in government health expenditures. There is a large gap in the scale of their health funding investment due to differences in the level of economic development, population size, and financial planning in various regions. According to the data in the China Health Statistics Yearbook in 2018, the government health expenditure of Guangdong Province (143.587 billion RMB), Henan Province (93.560 billion RMB), Shandong Province (RMB 91.710 billion), Sichuan Province (RMB 89.350 billion) and Jiangsu Province (RMB 868.95 billion) ranked in the top five, while Tianjin (RMB 21.633 billion), Hainan Province (RMB 14.760 billion), Qinghai Province (RMB 14.161 billion), Ningxia Hui Autonomous Region (RMB 10.838 billion), and Tibet Autonomous Region (RMB 11.128 billion) ranked the last five. Through these data. It is not difficult to find that in areas with small populations and underdeveloped levels of economic development, the scale of government investment in health funds is limited. Therefore, although the overall level of government health expenditure is relatively high, it may still not be enough to offset the health expenditure induced poverty caused by low government health expenditure. Second, the structure of government health expenditures does not match the needs of residents for medical services. Although the scale of medical financial expenditure is gradually increasing, if the overall demand for medical services is ignored, it will lead to an increase in personal health expenditure and further the risk of health expenditure induced poverty. Although government health expenditures continue to grow, drug prices are excessive due to poor market supervision, illegal operations by pharmaceutical companies, and medical staff inducing demand. Furthermore, patients overseeking also contributes to the rise of personal health expenditure. Zhang Dunfu and others also pointed out in their research that per capita medical and health expenditure will increase by 0.436% for every 1% increase in Chinese government health expenditure [53]. Third, government health expenditure may have a “diminishing marginal” effect on health. With the increase in people’s income, the gap in basic medical needs decreases. Hence, the marginal force of government health expenditure to relieve income constraints is reduced. The promotion effect on residents’ health care consumption is also weakened. Thus, the health expenditure poverty of the population is higher. Several studies might confirm our conclusions. An empirical study by Jie Chen et al. (2011) on residents’ health care expenditure in Jiangsu Province found that government health expenditure had a restraining effect on urban residents’ health care consumption [54]. Jie Mao et al. (2017) adopted an instrumental variable estimation method and found that the Chinese government’s public health investment in rural areas stimulates the growth of rural residents’ nonmedical consumption, but regrettably, it reduces rural residents’ medical consumption [55]. Data from China’s Health Statistics Yearbook show that government expenditure on health increased from RMB 52.356 billion to RMB 1520.587 billion, a 28-fold increase, from 1997 to 2017. Its share in government expenditure increased from 5.67 to 7.48%, and the parts in GDP rose from 0.66 to 1.84%. Both the absolute amount and the relative amount increased by a large margin. The per capita medical and health care consumption of urban residents increased from RMB 179.68 in 1997 to RMB 1777.4 in 2017, an increase of nearly 8.9 times. The share of health care in total consumption increased from 4.29 to 7.27%. The per capita medical and health care consumption of rural residents grew from RMB 62.45 to RMB 1058.7, an increase of nearly 16 times. Similarly, the proportion of health care consumption in total consumption expenditure went from 3.86 to 9.66%, more than quadrupling in 20 years. From these data, we consider that the growth of government investment in the medical field can improve the accessibility of medical resources for the masses and alleviate the “expensive” and “difficult” problems of medical treatment to a certain extent. However, government health expenditure may have a marginally diminishing effect on residents’ health expenditure poverty, with changes in people’s income level and health concepts.

Medical assistance, a crucial mode of social assistance, is considered the last line of defense to protect residents from poverty on health costs. However, we find that only one variable *X*_17_ passed the significance test in the dimension of medical assistance. We believe that this may be caused by insufficient medical assistance in China. Some studies can verify our conclusion. Yin Hang et al. (2017) analyzed the basic characteristics of families receiving medical assistance and their satisfaction with it. They found that the overall low level and limited assistance for families with serious illnesses are still problems in the current medical assistance in some parts of China [56]. Zhiqiang Jiang (2018) believed that medical assistance policies should be guided by expanding access and enhancing accuracy [57]. Additionally, Chan Sun (2020) pointed out that China’s medical assistance has problems such as an unbalanced financial expenditure structure, and poor families with expenditures are blind areas of assistance [58]. Therefore, all regions in China should actively explore a new mode of medical assistance that is fairer, more effective and covers more areas.

## Limitations

There are some shortcomings in this paper. Since this paper takes health expenditure poverty and the index of health services equity in different regions of China as the dependent and independent variables, it is inevitable that the sample size is small. Although the FGT poverty index can decompose the breadth, depth and strength of poverty distribution and compensate for the shortcomings of the traditional poverty index, it still only measures poverty from the single dimension of income. In the future, we will try to select other variables, expand the sample size, and use the multidimensional health poverty index to measure poverty to compensate for the existing deficiencies.

## Conclusion

This paper innovatively combined equity in health services utilization, equity in health financing, equity in health resources distribution and equity in the disadvantaged groups to establish the index system of health service equity as the independent variable. Then, the health expenditure poverty index of 26 regions in China was calculated as the dependent variable. The shortcomings of traditional linear regression, ridge regression and lasso regression are avoided by constructing elastic net regression model. In addition, this paper also used Geoda software to conduct spatial autocorrelation analysis on health expenditure poverty in different regions of China, which enriches the research on health expenditure poverty with spatial methods.

Comprehensive analysis of the full text shows that health service equity can alleviate poverty of health expenditure. In the process of strengthening the fairness of primary medical and health services, China should rationally allocate health resources and improve the accessibility of health services. Meanwhile, China needs to pay special attention to the spatial distribution of health investment, to reduce the risk of poverty in health expenditure caused by the unbalanced structure of government health expenditure. At present, China’s social security system mainly includes social insurance and medical assistance. Social insurance provides economic security for the majority of the population, so medical assistance should be the main force in alleviating health cost poverty. Therefore, the Chinese government should guarantee the financial needs of medical assistance and make good use of the power of social charity funds in poverty  alleviation. In addition, China needs to strengthen the implementation of policies related to the hierarchical diagnosis and treatment system and make the actual effect traceable to help residents in various regions share the achievements of the hierarchical medical system more fairly.

## Supplementary Information

**Additional file 1.**

## Data Availability

The datasets supporting the study are publicly available on the CFPS website http://www.isss.pku.edu.cn/cfps/ and China Health Statistics Yearbook. The specific situation has been explained in the text.
