# Supplementary material for: Can health service equity alleviate the health expenditure poverty of Chinese patients? Evidence from the CFPS and China health statistics yearbook
Source: BMC Health Serv Res. 2021 Jul 21;21:718. doi: 10.1186/s12913-021-06675-y (PMC8293547; doi:10.1186/s12913-021-06675-y)
Supplement: Supplementary file 1 — Additional file 1. [file 12913_2021_6675_MOESM1_ESM.docx]

**Can Health Service Equity Alleviate Health Expenditure Poverty of Chinese Patients?**

**Evidence from the CFPS and China Health Statistics Yearbook**

Shaoliang Tang^1^, Ling Yao^1^, Chaoyu Ye^1^, Zhengjun Li^1^, Jing Yuan^1^, Kean Tang^2^, David Qian^3^

1 School of Health Economics and Management, Nanjing University of Chinese Medicine, Nanjing, China

2 Faculty of Science, Skane, Lund University, Sweden

3 Swinburne Business School, Swinburne University of Technology, Australia

**【Correspondence】**

Shaoliang Tang, tangshaoliang@126.com

**Appendix**

**1. Principle of Global Moran’s I test**

Global Moran’s I measure the spatial autocorrelation between adjacent regions in the whole region. Its value is between 1 and -1, and 0 is used as the cut-off point to judge the positive correlation and negative correlation. If it is greater than 0, it means a positive correlation. Otherwise, it means a negative correlation. If the value is equal to 0, it means that the observed values are randomly distributed or there is no spatial autocorrelation. The more the value tends to 1 or -1, the stronger the aggregation of the same or opposite attributes is^[52]^. The Global Moran’s I test decides whether to reject the null hypothesis through the P value and the Z score. The P value represents the reliability of the data set, while the Z score and the value of Global Moran’s I indicate that the data set has obvious aggregation or dispersion law. The Global Moran’s I is expressed as:

$Globle Molan^{'}s I=\frac{\sum_{i=1}^{n} \sum_{j=1}^{n} W_{ij}(x_{i}-\bar{x})(x_{j}-\bar{x})}{S^{2}S_{0}}$ (1)

$\bar{x}=\frac{1}{n}\sum_{i=1}^{n} x_{i}$ (2)

$S^{2}=\frac{1}{n}\sum_{i=1}^{n} (x_{i}-\bar{x})$ (3)

$S_{0}=\sum_{i=1}^{n} \sum_{j=1}^{n} W_{ij}$ (4)

In these formulas, $x_{i}$ is the value of variable {$x_{i}$}in position or region $i$. $\bar{x}$ represents the mean value of the variables. $S^{2}$ is on behalf of the variance of the variable. $S_{0}$ is the sum of spatial weights of all variables. $n$ means the total number of observations of the variable, in other words, the total number of regions or locations corresponding to the observations. $W_{ij}$ is an element in the spatial weight matrix $W$, which refers to the spatial weight between region or position $i$ and $j$. If the spatial unit $i$ is set to be adjacent to $i$ , $W_{ij}=1$; Otherwise $W_{ij}=0$ ^[52]^.

In addition, we need to test whether the Global Moran's I is statistically significant. When the number of regions n is large enough, Moran index approximately obeys normal distribution. As a result, we can use Z test to verify it.

$Z=\frac{I-E(I)}{\sqrt{var（I）}}$ (5)

**2.** **Principle of Local Moran’s I test**

Once the Global Moran’s I test shows autocorrelation, we can locate the specific location of outliers or aggregation through the Local Moran’s I test^[52]^. Different from the Global Moran’s I, the Local Moran’s I has no limit and is not limited to [-1,1]. Local Moran’s I greater than 0 reveals that the high (low) value of region $i$ is surrounded by the surrounding high (low) values, and a value less than 0 indicates that the high (low) value of region $i$ is surrounded by the surrounding low (high) values. The formula of Local Moran’s I is as follows.

${Local Molan^{'}s I}_{i}=\frac{Z_{i}}{S^{2}}\sum_{j\neq i}^{n} W_{ij}Z_{ij}$ (6)

Where,${Local Molan^{'}s I}_{i}$represents the Local Moran's I value of $i$ region.$Z_{i}=y_{i}-\bar{y}$ , $Z_{j}=y_{j}-\bar{y}$ and $S^{2}=\frac{1}{n}\sum{(y_{i}-\bar{y})}^{2}$ are the spatial weight value, and $n$ is the total number of all regions.

Reference

1. Fang XU, Wei Liu. The Determinations of Chinese Urban Residents Health Care Expenditure: A Cointegration Approach[J]. China Population, Resources and Environment, 2014,24(S1):239-243.
